# Supplementary material for: The DNA adenine methylase of Salmonella Enteritidis promotes their intracellular replication by inhibiting arachidonic acid metabolism pathway in macrophages
Source: Front Microbiol. 2023 Mar 2;14:1080851. doi: 10.3389/fmicb.2023.1080851 (PMC10018194; doi:10.3389/fmicb.2023.1080851)
Supplement: Supplementary file 2 [file Data_Sheet_2.zip › S1 Appendix. Non-targeted metabolomics raw data/4.MetDiffAnalysis/C50336_WT.vs.control/C50336_WT.vs.control_neg_heatmap2.pdf]

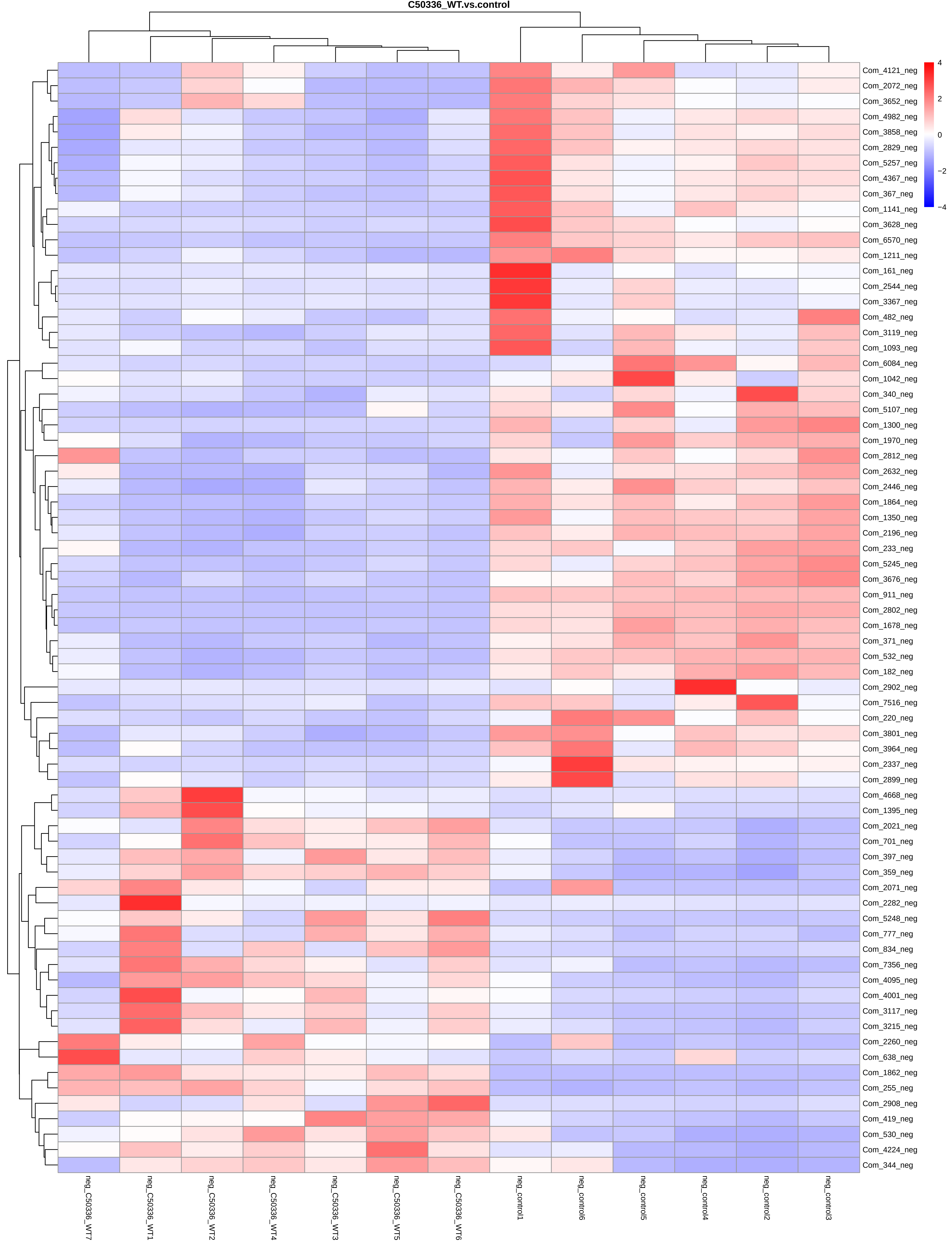

C50336_WT.vs.control
4
2
0
−2
−4
Com_4121_neg
Com_2072_neg
Com_3652_neg
Com_4982_neg
Com_3858_neg
Com_2829_neg
Com_5257_neg
Com_4367_neg
Com_367_neg
Com_1141_neg
Com_3628_neg
Com_6570_neg
Com_1211_neg
Com_161_neg
Com_2544_neg
Com_3367_neg
Com_482_neg
Com_3119_neg
Com_1093_neg
Com_6084_neg
Com_1042_neg
Com_340_neg
Com_5107_neg
Com_1300_neg
Com_1970_neg
Com_2812_neg
Com_2632_neg
Com_2446_neg
Com_1864_neg
Com_1350_neg
Com_2196_neg
Com_233_neg
Com_5245_neg
Com_3676_neg
Com_911_neg
Com_2802_neg
Com_1678_neg
Com_371_neg
Com_532_neg
Com_182_neg
Com_2902_neg
Com_7516_neg
Com_220_neg
Com_3801_neg
Com_3964_neg
Com_2337_neg
Com_2899_neg
Com_4668_neg
Com_1395_neg
Com_2021_neg
Com_701_neg
Com_397_neg
Com_359_neg
Com_2071_neg
Com_2282_neg
Com_5248_neg
Com_777_neg
Com_834_neg
Com_7356_neg
Com_4095_neg
Com_4001_neg
Com_3117_neg
Com_3215_neg
Com_2260_neg
Com_638_neg
Com_1862_neg
Com_255_neg
Com_2908_neg
Com_419_neg
Com_530_neg
Com_4224_neg
Com_344_neg
neg_C50336_WT7
neg_C50336_WT1
neg_C50336_WT2
neg_C50336_WT4
neg_C50336_WT3
neg_C50336_WT5
neg_C50336_WT6
neg_control1
neg_control6
neg_control5
neg_control4
neg_control2
neg_control3
